# Disruption of *AtWNK8* Enhances Tolerance of *Arabidopsis* to Salt and Osmotic Stresses via Modulating Proline Content and Activities of Catalase and Peroxidase

**DOI:** 10.3390/ijms14047032

**Published:** 2013-03-27

**Authors:** Baige Zhang, Kaidong Liu, Yan Zheng, Yingxiang Wang, Jinxiang Wang, Hong Liao

**Affiliations:** 1 State Key Laboratory for Conservation and Utilization of Subtropical Agro-bioresources, Root Biology Center, College of Natural Resources and Environment, South China Agricultural University, Guangzhou 510642, China; E-Mails: plantgroup@126.com (B.Z.); liukaidong@zhjnc.edu.cn (K.L.); zysktyy@hotmail.com (Y.Z.); yxwang@scau.edu.cn (Y.W.); hliao@scau.edu.cn (H.L.); 2 Vegetable Research Institute, Guangdong Academy of Agricultural Sciences, Guangzhou 510640, China; 3 Life science and Technology School, Zhanjiang Normal University, Zhanjiang 524048, China; 4 State Key Laboratory of Genetic Engineering, Institute of Plant Biology, School of Sciences, Fudan University, 220 Handan Road, Shanghai 200433, China

**Keywords:** *AtWNK8*, *Arabidopsis*, salt stress, osmotic stress, proline, catalase, peroxidase

## Abstract

With no lysine kinases (WNKs) play important roles in plant growth and development. However, its role in salt and osmotic stress tolerance is unclear. Here, we report that *AtWNK8* is mainly expressed in primary root, hypocotyl, stamen and pistil and is induced by NaCl and sorbitol treatment. Compared to the wild-type, the T-DNA knock-out *wnk8* mutant was more tolerant to severe salinity and osmotic stresses, as indicated by 27% and 198% more fresh weight in the NaCl and sorbitol treatment, respectively. The *wnk8* mutant also accumulated 1.43-fold more proline than the wild-type in the sorbitol treatment. Under NaCl and sorbitol stresses, catalase (CAT) activity in *wnk8* mutant was 1.92- and 3.7-times of that in Col-0, respectively. Similarly, under salt and osmotic stress conditions, peroxidase (POD) activities in *wnk8* mutant were 1.81- and 1.58-times of that in Col-0, respectively. Taken together, we revealed that maintaining higher CAT and POD activities might be one of the reasons that the disruption of *AtWNK8* enhances the tolerance to salt stress, and accumulating more proline and higher activities of CAT and POD might result in the higher tolerance of *WNK8* to osmotic stress.

## 1. Introduction

Salinity generates both ionic and osmotic stresses in plants [1]. Ionic stress is caused by toxic levels of sodium (Na^+^) in the cytoplasm and by deficiencies of other ions, such as K^+^[2]. Under both salt and osmotic stress conditions, increased generation of H_2_O_2_ (hydrogen peroxide), O_2_^−^ (superoxide), O_2_ (singlet oxygen) and OH (hydroxyl) radicals enhance leakage of electrons to molecular oxygen [1,3]. These cytotoxic reactive oxygen species (ROS) can destroy normal metabolism through oxidative damage to lipids, proteins and nucleic acids, leading to cell membrane damage and malondialdehyde (MDA) production [4]. Accordingly, plants have evolved an oxygen-scavenging system, which consists of superoxide dismutase (SOD, EC 1.15.1.1), catalase (CAT, EC 1.11.1.6), peroxidase (POD, EC 1.11.1.7), and so on [5].

Under drought-induced osmotic conditions, plant roots sense water deficit signals and produce more abscisic acid (ABA), which is transported to leaves through vascular tissue, leading to stomata closure to reduce water loss [3]. One of the best characterized biochemical responses of plant cells to osmotic stress is the accumulation of organic osmolytes, such as proline and betaines [6]. These substances, in turn, stabilize membranes and maintain protein conformation at low leaf water potential [7].

Protein kinases have been reported to regulate responses of plants to salt and osmotic stresses. For instance, Ca^2+^ signals, triggered by salt stress, are perceived by SOS3—the binding of calcium and myristoylation of SOS3 are necessary for SOS3 function—and then, SOS3 activates the SOS2 kinase; the subsequently activated SOS2 kinase phosphorylates the SOS1 Na^+^/H^+^ antiporter, which then pumps Na^+^ out of the cytosol [8].

With no lysine kinase (WNK) is a subfamily of serine/threonine protein kinases existing in both animals and plants. A lysine residue in subdomain II of kinase is very important for Adenosine triphosphate (ATP) binding and highly conserved among all the other kinase subfamilies, which is missed in this WNK subfamily, but replaced by another lysine in subdomain I [9,10]. Human WNKs are genetically linked to the regulation of blood pressure [11]. However, WNKs are not found in yeast, indicating WNKs are restricted to multicellular organisms [12]. However, the roles of WNKs in plants are poorly understood.

In *Arabidopsis*, 10 *WNK* genes (*AtWNK*1-10) are supposed to encode WNKs, whereas the rice genome contains seven *WNK*s *(OsWNK*1-7) [13]. Compared with mammalian WNKs, plant WNKs are much smaller proteins with a predicted molecular weight of about 60–70 KDa. With the exception of *AtWNK1* and *OsWNK5*, which consist only of a highly conserved *N*-terminal kinase domain, all the other plant *WNK*s have a highly divergent *C*-terminal domain of about 300 amino acids [13]. *AtWNK1* phosphorylates the APRR3 member of the APRR1/TOC quintet, which is involved in the circadian rhythm [14]. On the other hand, the transcription of *AtWNK2*, *AtWNK4* and *AtWNK6*, together with *AtWNK1*, are controlled by circadian rhythm [14]. AtWNK8 (At5g41990), a member of the WNK family, interacts with subunit C of the vacuolar H^+^-ATPase *in vitro* via a special short *C*-terminal domain and phosphorylates *Arabidopsis* vacuolar H^+^-ATPase subunit C at multiple sites [15], implicating that *AtWNK8* is a participant in stress responses. AtWNK8 is localized in the nucleus and interacts physically with *Arabidopsis* enhanced downy mildew 2 (EDM2), which appears to act downstream of AtWNK8 in controlling floral transition by modulating expression of the floral repressor gene, flowering locus C (FLC, At5g10140) [16]. Recently, a soybean root-specific *WNK* homolog, *GmWNK1*, had been identified to regulate root development, possibly through mediating ABA homeostasis *in vivo*[17]. Hence, we hypothesized that *AtWNK8* might mediate *Arabidopsis* responses to osmotic and salt stresses.

To date, the roles of *AtWNK8* in abiotic stress responses remain unclear. In this study, we employed a T-DNA insertion mutant of *WNK8* and an overexpression line of *WNK8* (*WNK8-OE*) to study the roles of *WNK8* in salt and osmotic stresses at physiological and molecular levels. We revealed that disruption of *AtWNK8* enhances tolerance of *Arabidopsis* to salt and osmotic stresses mightbe via modulating proline content and activity of CAT and POD.

## 2. Results and Discussion

### 2.1. Activities of the AtWNK8 Promoter at Tissue Level

**Figure 1:**
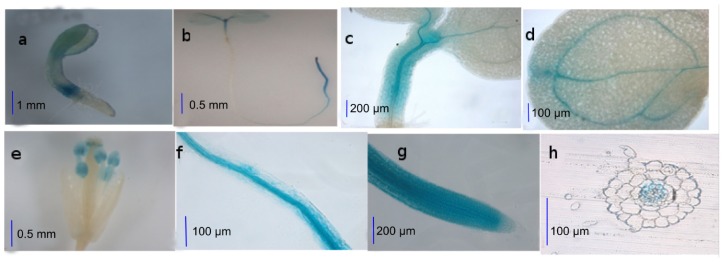
Expression patterns of the *AtWNK8* gene. *Arabidopsis* Col-0 was transformed with a binary vector containing a pro*AtWNK8::GUS* construct. Homogenous T3 transgenic plants were stained with X-gluc for 12 h, following the standard methods. The activities of *AtWNK8* promoter were detected in two-day-old (**a**) or seven-day-old (**b**–**d**, **f**–**h**) or four-week-old (**e**) *Arabidopsis*, as illustrated: (**a**) two-day old seedling; (**b**) seven-day-old seedling; (**c**) hypocotyl; (**d**) leaves; (**e**) flowers; (**f**) root elongation zone; (**g**) root tip; and (**h**) cross section of root tip.

To investigate temporal and spatial activities of the *AtWNK8* promoter, we analyzed beta-glucuronidase (GUS) staining at the tissue level using transgenic *Arabidopsis* harboring the promoter of *AtWNK8* and a fused GUS reporter gene. The histochemical GUS staining results revealed that the promoter of *AtWNK8* was active in stem and cotyledon of two-day-old seedlings, especially in the junction of hypocotyl and primary root (PR) (Figure 1a). In seven-day-old seedlings, a GUS signal was detected in roots, hypocotyls and leaves (Figure 1b). Results in Figure 1c, d indicated that the promoter of *AtWNK8* was mainly active in hypocotyls near shoot apex and veins of cotyledon. GUS signal was also high in stamens and pistils of four-week-old plants (Figure 1e). Figure 1f, g showed the activity of *WNK8* promoter in roots, except root cap. Furthermore, root cross-section showed that the activity of *WNK8* promoter was mainly confined in vascular tissues (Figure 1h). In short, the *WNK8* promoter is universally active in the hypocotyls, roots, stems and flowers, indicating that *WNK8* appears to play important roles in plant growth and development.

Bioinformatics analysis (http://arabidopsis.med.ohio-state.edu, [18]) revealed that the promoter of *WNK8* might have several transcriptional factor (TF) binding elements, such as a putative DRE binding site, CCGAC [19], located in −1546 and −1541, a cold response factor binding site, ACTCCG [20], at −236 position, and GT-1 binding sites, GA(/G)A(T)AAA(/T) [21], at −274, −979 and −1366 (Table 1), suggesting that the transcript level of *WNK8* might be regulated by stress-related TFs.

### 2.2. Induction of AtWNK8 by NaCl and Sorbitol Stresses in *Arabidopsis*

In mammals, *WNK*s are ion or osmotic stress-responsive genes [22]. To clarify whether *AtWNK8* transcription is induced by salt or osmotic stresses, *Arabidopsis* seedlings were subjected to a high concentration of NaCl or sorbitol-generated stress. Quantitative real-time polymerase chain reaction (qRT-PCR) was further employed to analyze the expression of *AtWNK8*.

**Figure 2:**
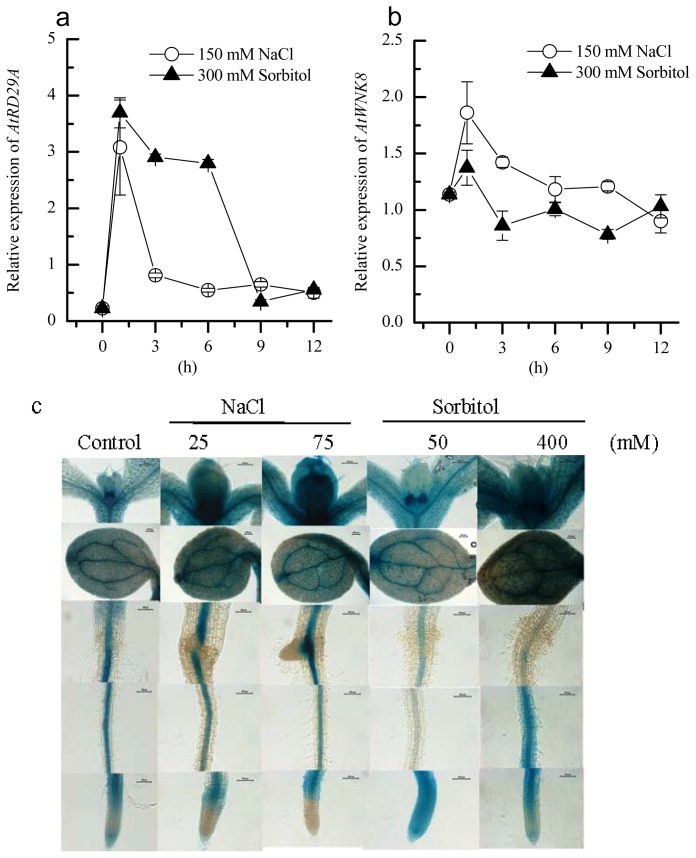
Responses of the *AtWNK8* expression to salt and osmotic stresses. The expression levels of *AtRD29A* (**a**) and *AtWNK8* (**b**) were determined by quantitative real-time polymerase chain reaction (qRT-PCR), and the beta-glucuronidase (GUS) signal in transgenic *Arabidopsis* harboring *proWNK8::GUS* was observed through standard methods (**c**). Total RNA was extracted from whole seedlings. Seven-day-old seedlings were treated with NaCl or sorbitol, as the indicated time (**a** and **b**), or treated with NaCl or sorbitol for 12 h (**c**); Scale bar, 100 μm. Data were the means ± SE of three independent experiments.

Considering *RD29A* (*At5g52310*) is a stress responsive marker gene, it is induced by drought, salinity, cold and ABA [23]. We firstly detected the expression of *AtRD29A* with RNA samples from whole seedlings. Results in Figure 2a indicated that *RDA29A* was induced very quickly by NaCl and sorbitol treatment and then decreased. Whereas the *WNK8* expression was induced rapidly and peaked at 1 h after high NaCl and sorbitol treatments, the duration of higher *WNK8* expression level was short. The *AtWNK8* expression gradually decreased to a normal level at 12 h after treatment (Figure 2b). Moreover, salt stress more strongly induced *WNK8* expression than sorbitol at 1 h after treatment. Generally, the responsive patterns of *WNK8* were similar to *RD29A* (Figure 2a,b). To further determine the responses of *AtWNK8* to the above-mentioned stresses at tissue level, the transgenic seedlings were treated with two concentrations of NaCl and sorbitol for 12 h. As shown in Figure 2c, under control conditions, the GUS staining can be detected in roots, leaves, stems and shoot apexes. The activities of the *WNK8* promoter were induced in flowers and leaves, as well as the upper part of primary roots (PRs) under salt stress. In addition, 50 mM sorbitol treatment only stimulated the activities of *WNK8* promoter in the root tips, but 400 mM sorbitol upregulated the activities of *WNK8* promoter in the aerial part, root tips and elongation zone of PRs. Whereas a previous study showed that the transcripts of *WNK8* were higher in roots at the seedling stage (two-week-old) and in stems at the flowering stage (six-week-old), they were almost undetectable in other organs based on RT-PCR results [13]. Altogether, data in Figure 2 indicated that *AtWNK8* responded to the changes of environmental NaCl and sorbitol concentration.

Dehydration, cold, salt and osmotic stresses, as well as ABA induced the expression of *RD29A*[23]. The 120 bp promoter region of *RD29A* contains the DRE, DRE/CRT-core motif (A/GCCGAC) and ABRE element [23]. Consistently, the *WNK8* promoter contains a DRE- and cold-responsive *cis*-element (Table 1). It is well known that salt and osmotic stresses result in dehydration, so we reasoned that salt and osmotic stress-induced *WNK8* expression seems to be related to DRE elements. Furthermore, identification of *WNK8* promoter-binding TFs would facilitate deciphering functions of *WNK8* in salt and osmotic stress responses.

### 2.3. Responses of Knock-Out Mutant wnk8 and Overexpression Line of *WNK8* (*WNK8-OE*) to Salt and Osmotic Stresses

To obtain further evidence of the *in vivo* functions of *WNK8* responding to salt and osmotic stresses, initially we employed two T-DNA insertion mutants (SALK_024887 and SALK_058925; see [13]) and two independent *WNK8* overexpression lines (*WNK8-OE*) to explore the performance of those lines under 150 mM NaCl and 300 mM sorbitol. Moreover, we found that the two independent T-DNA insertion lines or two overexpression lines showed similar responses under the two adverse conditions (data not shown), so we just employed one T-DNA insertion line (SALK_024887) as *wnk8* mutant and one overexpression line as *WNK8-OE* in the follow-up experiments, such as measuring the contents of chlorophyll (Chl), relative water content (RWC), Fresh weight (FW), proline content and activities of POD and CAT.

The loss of the function mutants in *WNK8* (SALK_024887 and SALK_058925) were characterized by previous studies [13], and the overexpression lines (*WNK8-OE*) were generated (see Experimental Section and Figure S1). PCR analysis verified that the homozygous *AtWNK8* T-DNA insertion mutant *wnk8* was a 100% knock-out mutant that did not transcribe the *WNK*8 gene (data not shown, see [13]). qRT-PCR analysis showed that the expression level of *WNK8* was over three-fold higher in the homozygous T3 *WNK8-OE* line than that in the wild-type Col-0 (Figure S1).

**Figure 3:**
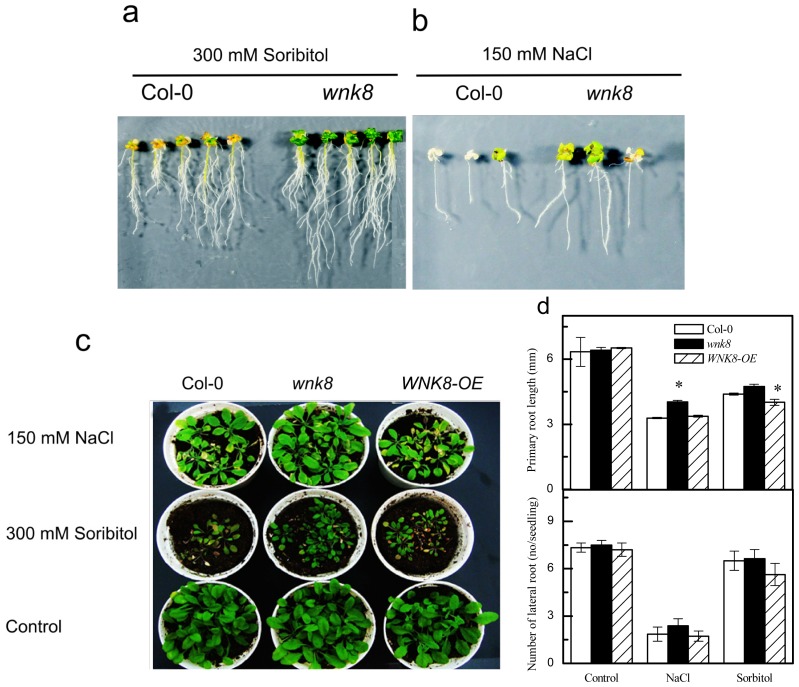
The *wnk8* mutant was more tolerant to salt and osmotic stresses. Seeds from wild-type and *WNK8* plants were germinated on standard 1/2 MS medium for three days; then, uniform seedlings were transferred to vertically placed plates containing 300 mM sorbitol for 30 days (**a**) or 150 mM NaCl for 10 days (**b**). Three-day-old seedlings of Col-0, *wnk8* and *WNK8-OE* grown in standard 1/2 MS medium were transferred to soils and grown for 20 days and then treated with 150 mM NaCl or 300 mM sorbitol via top irrigation once every three days. Photos were taken after a 15-day treatment (**c**). Stratified seeds were directly germinated in 1/2 MS media containing 150 mM NaCl or 300 mM sorbitol for seven days in vertically placed petri dishes, and then, the length of PR and the number of lateral roots were counted (**d**). Data are the means ± SE from three independent experiments, and each experiment included 20 seedlings tested. Asterisks on bars indicate significant differences in contrast to Col-0 under the same treatment (Student’s *t*-test, *p* < 0.05).

Grown in 300 mM sorbitol (Figure 3a) for 30 days or 150 mM NaCl (Figure 3b) for 10 days, the wild-type plants progressively turned yellow, exhibited chlorosis and ceased growth, but the *wnk8* mutant plants remained relatively green and grew slowly on petri dishes, suggesting that *WNK8* is a negative player for salt and osmotic stress adaptations.

Next, to investigate whether the altered stress responses occurred in natural conditions, the wild-type Col-0, *wnk8* and *WNK8-OE* seedlings were planted in soil. Figure 3c shows that under the two stresses, progressive chlorosis, reduced leaf size and general growth inhibition of wild-type and *WNK8-OE* were observed after all treatments through top irrigation and eventually died. In contrast, the *wnk8* mutant survived longer. Compared to sorbitol treatment, NaCl treatment had a relatively smaller adverse effect on *wnk8* growth. In addition, the *wnk8* mutant also showed more tolerance to mannitol treatment (data not shown). On the other hand, the difference of the tolerance to NaCl was not as obvious as the tolerance to sorbitol among Col-0, *wnk8* and *WNK8-OE.* This indicated that *WNK8* plays more negative roles in the responses to osmotic stress than to salt stress.

Numerous studies demonstrated that root system architecture is dramatically influenced by environmental factors, including salt and osmotic stresses [24,25]. To investigate the involvement of *AtWNK8* in root growth and development as affected by the two adverse conditions, uniform seedlings were grown in 1/2 MS media containing 150 mM NaCl or 300 mM sorbitol for seven days in vertically placed petri dishes. The length of PR and the number of lateral roots (LRs) were measured. As shown in Figure 3d, both NaCl and sorbitol applications significantly decreased PR length and the number of LRs in Col-0, *wnk8* and *WNK8-OE*, whereas the PR length of *wnk8* was longer than Col-0 and *WNK8-OE* under salt stress, and the PR length of *WNK8-OE* was shorter than Col-0 and *wnk8* under osmotic stress. This indicated that *WNK8* negatively regulated PR growth under the two stressed conditions. For the number of LRs, no significant differences among Col-0, *wnk8* and *WNK8-OE* were found under normal and adverse conditions (Figure 3d). These data implied that *WNK8* appeared to play a small role in LR development.

### 2.4. Effects of Salt and Osmotic Stresses on Fresh Weight (FW), Relative Water Content (RWC) and Chlorophyll Content of Col-0, wnk8 Mutant and *WNK8-OE* Plants

**Figure 4:**
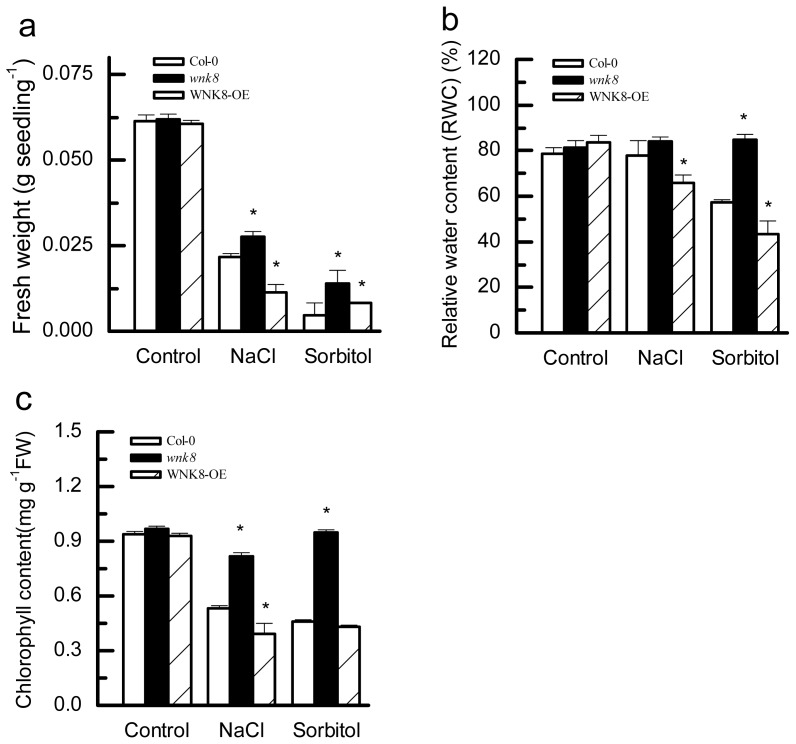
Fresh weight (FW) (**a**), relative water content (RWC) (**b**) and chlorophyll content (**c**) in Col-0, *wnk8* mutant and *WNK8-OE* under salt and osmotic stress conditions. (**a**) Three-day-old seedlings germinated in standard 1/2MS media were transferred to 150 mM NaCl or 300 mM sorbitol for treatment. FW was measured after seven days of treatment. Data are the means ± SE from three independent experiments, and each experiment included 20 seedlings tested. Asterisks on bars indicate significant differences in contrast to the wild-type under the same treatment (Student’s *t*-test, *p* < 0.05). (**b**,**c**) Three-day-old seedlings grown in 1/2 MS media were transferred to soil and grown for 20 days and then treated with 150 mM NaCl or 300 mM sorbitol via irrigation once every three days. After a 15-day treatment, RWC (**b**) and chlorophyll content (**c**) were measured. Data are the means ± SE from three independent experiments, and each experiment included 20 seedlings tested. Asterisks on bars indicate significant differences in contrast to the wild-type under the same treatment (Student’s *t*-test, *p* < 0.05).

As shown in Figure 4a, under normal growth conditions, there were no significant differences among Col-0, *wnk8* and *WNK8-OE*, as indicated by FW. By contrast, salt and osmotic stresses significantly inhibited growth of Col-0, *wnk8* and *WNK8-OE*, resulting in less FW. Under salt stress conditions, the FW of *WNK8* was the highest, followed by Col-0 and *WNK8-OE*, and the FW of *WNK8* was 1.27-times that of Col-0 (*p* < 0.05). Under osmotic stress conditions, the FW of *wnk8* was 2.98-times that of Col-0 (*p* < 0.05).

Results in Figure 4b showed that as to RWC, no significant differences among Col-0, *wnk8* and *WNK8-OE* were found under control conditions. However, under salt stress, RWC of *WNK8-OE* was significantly lower than *wnk8* and Col-0. More importantly, subjected to osmotic stress, the RWC of *wnk8* was higher than that of Col-0; consistently, the RWC of *WNK8-OE* was lower than that of Col-0 (*p* < 0.05).

Compared with the control, NaCl and sorbitol treatments significantly decreased the chlorophyll content of Col-0 and *WNK8-OE*, but not the *wnk8* mutant. Under salt and osmotic stress conditions, the chlorophyll content of *WNK8* was significantly higher than Col-0 and *WNK8-OE* (*p* < 0.05, Figure 4c).

These results indicated that *WNK8* was a negative regulator for *Arabidopsis* in response to salt and osmotic stresses. The relatively high level of RWC and chlorophyll content of *WNK8* under the two adverse conditions will maintain cell activity and stabilize photosynthesis, thus leading to more FW. Taken together, the *WNK8* mutant appeared to be less sensitive, but more tolerant, to salt and osmotic stresses in terms of physiological and growth parameters.

### 2.5. Higher Tolerance of wnk8 to Salt and Osmotic Stresses Was Partially Achieved by Accumulating More Proline

**Figure 5:**
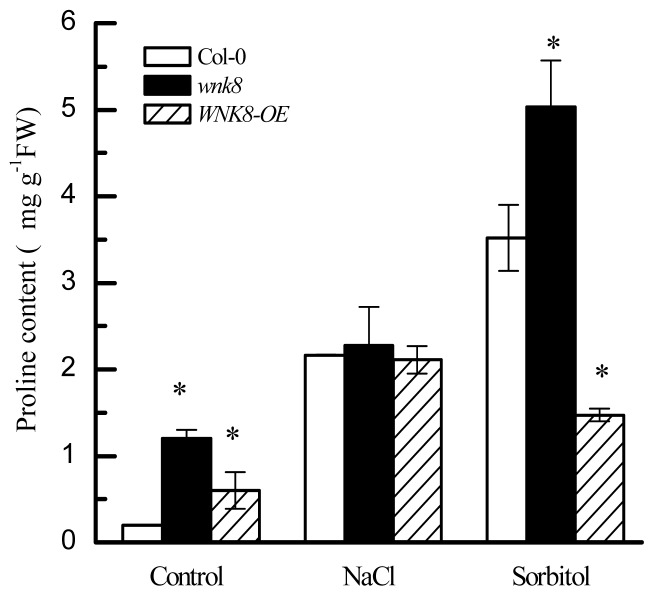
Proline content in Col-0, *wnk8* and *WNK8-OE* under salt and osmotic stress conditions. Three-day-old seedlings grown in standard 1/2 MS media were transferred to soil and further cultivated for 20 days and then treated with 150 mM NaCl or 300 mM sorbitol via irrigation once every three days. After 15 days, the proline content was measured. Data are the means ± SE from three independent experiments, and each experiment included 20 uniform seedlings tested. Asterisks on bars indicate significant differences in contrast to the wild-type under the same treatment (Student’s *t*-test, *p* < 0.05).

Apart from acting as a compatible solute to protect cells from dehydration damage, proline can act as a free radical scavenger, thus increasing plant tolerance to abiotic stresses [26]. Figure 5 showed that Col-0, *wnk8* and *WNK8-OE* plants all accumulated proline under salt and osmotic stresses, implying that accumulation of proline is a universal mechanism of *Arabidopsis* to cope with the two abiotic stresses.

Under control conditions, the content of proline in *wnk8* and *WNK8-OE* was higher than that in Col-0. Although salt stress stimulated proline accumulation in Col-0, the proline content was similar among Col-0, *wnk8* and *WNK8-OE* under salt stress. This suggested that alteration of *WNK8* transcription had no effect on proline accumulation under salt stress, and the increased tolerance of the *wnk8* mutant to salinity was independent of proline accumulation. More interestingly, under osmotic stress, the content of proline in *wnk8* was the highest and that in *WNK8-OE* was the lowest, indicating that the greater tolerance of *wnk8* to osmotic stress seems to be dependent on proline accumulation. It was documented that P5C synthase (P5CS) and P5C reductase catalyzed proline biosynthesis [27], and proline dehydrogenase (ProDH) and P5C dehydrogenase mediated proline degradation in plants [28]. Further studies are needed to clarify whether loss of function of *WNK8* altering the proline level in *Arabidopsis* is dependent on P5CS and P5C reductase under salt and osmotic stresses.

### 2.6. The wnk8 Mutant Maintained Higher Activities of CAT and POD in Salt and Osmotic Stresses

**Figure 6:**
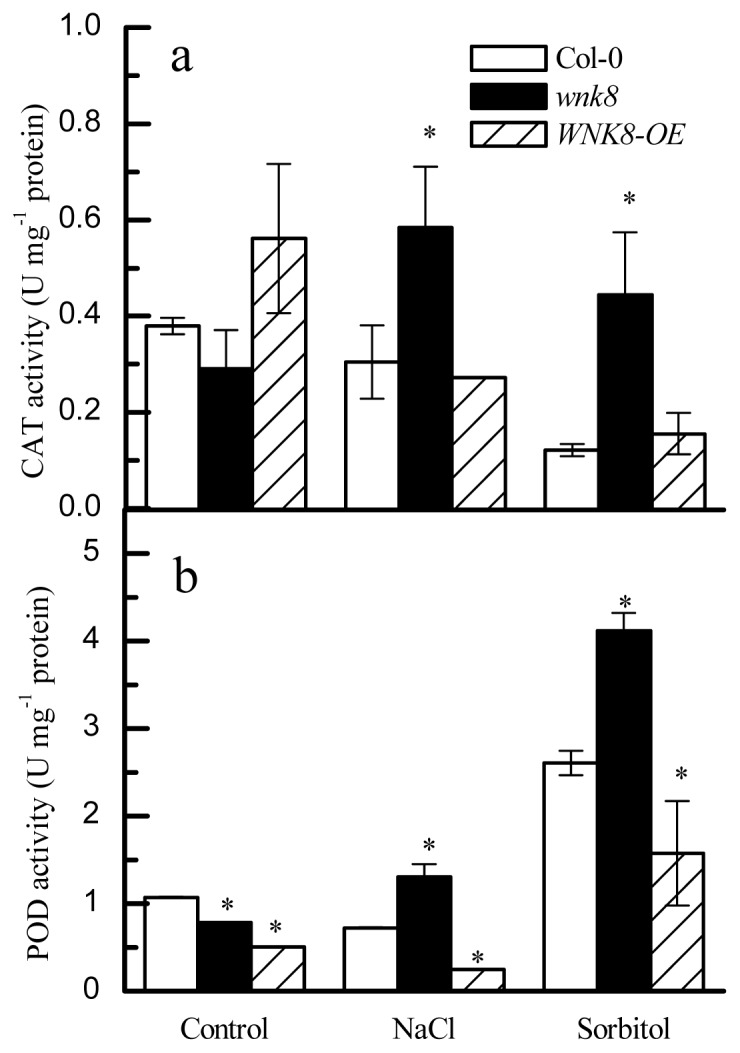
Activities of catalase (CAT) and peroxidase (POD) in Col-0, *wnk8* and *WNK8-OE* under salt and osmotic stress conditions. Three-day-old seedlings grown in standard 1/2 MS media were transferred to soil and grown for 20 days and then treated with 150 mM NaCl or 300 mM sorbitol via irrigation once every three days. After 15 days, the activities of CAT (**a**) and POD (**b**) were determined. Data are the means ± SE from three independent experiments, and each experiment included 20 uniform seedlings tested. Asterisks on bars indicate significant differences in contrast to the wild-type under the same treatment (Student’s *t*-test, *p* < 0.05).

The ability to scavenge ROS in a short time is crucial for plants to tolerate stress and survive. Antioxidative CAT and POD are important enzymes for quenching ROS in plants [29]. As shown in Figure 6a, under control conditions, no difference of CAT activity among Col-0, *wnk8* and *WNK8-OE* can be found. However, under salt stress, the *WNK8* mutant had 1.92-fold higher CAT activity than Col-0, but no difference between Col-0 and *WNK8-OE*. When subjected to sorbitol stress, the activity of CAT in *wnk8* mutant was 3.66-fold of that in the wild-type (*p* < 0.05), but the difference between Col-0 and *WNK8-OE* was insignificant (*p* > 0.05, Figure 6a). We noticed that both salt and sorbitol treatments significantly increased CAT activity in *WNK8*.

Figure 6b showed that under control conditions, the POD activity in Col-0 was significantly higher than that in *WNK8* and *WNK8-OE*, but opposite in salt stress; the POD activity in *wnk8* was 1.81-times higher than Col-0 and 5.33-times higher than *WNK8-OE*. On the other hand, under sorbitol stress, relative to Col-0, the POD activity in *wnk8* was increased by 58%, and that in *WNK8-OE* was significantly lower (*p* < 0.05). This indicated that *WNK8* is a negative regulator for POD activity under salt and osmotic stress conditions.

CAT and POD are involved in quenching ROS in plant tissues. Results in Figure 6 implied that maintaining higher CAT and POD activity might be one of the reasons why *wnk8* mutant was more tolerant to salt and osmotic stresses. Since AtWNK8 is a kinase, we cannot rule out that CAT and POD may be directly phosphorylated by it or indirectly mediated by WNK8 through other unknown proteins.

More interestingly, Urano *et al.* (2012) demonstrated that WNK8 phosphorylates AtRGS1 (regulator of G-protein signaling 1) and then stimulates the endocytosis of AtRGS1, and the induced AtRGS1 endocytosis by d-glucose, but not l-glucose, is dependent on WNK8 [30]. Accordingly, the *wnk8* mutant has decreased sensitivity to 6% d-glucose, and overexpression of WNK8 results in more sensitivity to d-glucose [30]. At this point, WNK8 seems to be not involved in glucose-generated osmotic stress responses, but is involved in glucose signaling. On the other hand, overexpression of GmWNK1 in *Arabidopsis* increases the tolerance to salt and osmotic stresses [31], and GmWNK1 is more close to AtWNK1 [17]. We noticed that AtWNK8 is most divergent form AtWNK1 [30]. Given the opposite effects of AtWNK1 and AtWNK8 in osmotic and salt stresses, it is tempting to reveal underlying mechanisms.

## 3. Experimental Section

### 3.1. Plant Materials and Growth Conditions

All the *Arabidopsis thaliana* plants used in this study were Columbia-0 (Col-0) ecotype. Genomic DNA was extracted via the cetyl trimethylammonium bromide (CTAB) method [32]. We amplified the promoter fragment of *AtWNK8* 1804 bp upstream of the ATG initiation code through PCR with the primer pair composed of the following forward primer (5′-AAAggatccCTCTGCTGCGTTCTTTGGG C-3′) and the reverse primer (5′-AAAccatggCAAACAAAGCAATCGAGAAC-3′). The pCAMBIA 1305.2 plasmid [33] DNA digested by BamHI and NcoI was cut with the original 35S promoter and ligated with the *AtWNK8* promoter digested by BamHI and NcoI enzyme and then transformed with the constructed pCAMBIA 1305.2 vector containing the *proWNK8::GUS* construct via the floral dip method [34]. Transformed lines were selected in hygromycin-containing medium. Homozygous T3 seedlings were used in the subsequent experiments.

Total RNA was extracted through a TRIZol method and subjected to reverse transcriptase polymerization to get cDNA. The forward primer (5′-AAAggatccgATGGCTTCTGGTTCTGGATT-3′) and the reverse primer (5′-AATtctagaAGAGATGTTAACTGCTTTTTGCT-3′) were used to clone the full length *WNK8* cDNA open reading frame (ORF) into pCAMBIA1380, which harbors a 35S promoter. Col-0 was transformed via the floral dip method [34] to get homozygous *WNK8-OE* lines.

Two independent *AtWNK8* T-DNA insertion lines (SALK_024887 and SALK_058925) were obtained from ABRC (Columbus, OH, USA). The homozygous T-DNA insertion lines were verified via RT-PCR, as described by Alonso *et al.* (2003) [35] and Wang *et al.* (2008) [13], using primers LBa1 (5′-CGGAACCACCATCAAACAGG-3′), *AtWNK8*-LP (5′-GTCCTTGCCTCCATCCCTTGCA-3′), and *AtWNK8*-RP (5′-GTCCTTGCCTCCATCCCTTGCA-3′).

### 3.2. NaCl and Sorbitol Treatments

For sterilized media growth, seeds of wild-type, *wnk8*, and *WNK8-OE* were surface sterilized with 70% ethanol for 1 min and then with 10% bleach for 5 min before being washed five times with sterilized water. The seeds were germinated and grown on vertically placed petri dishes containing sterilized half strength MS media (pH 5.7), as described in Wang *et al.* (2008) [13]. Uniform 3-day-old plants were transplanted into 1/2 MS media supplemented with NaCl or sorbitol at indicated concentrations and grown for the indicated time. For taking pictures to indicate the differences between lines, seedlings were treated with NaCl for 10 days or with sorbitol for 30 days.

For measuring root parameters, stratified seeds were grown in 1/2 MS media containing 150 mM NaCl or 300 mM sorbitol for 7 days in vertically placed petri dish before being scanned. The lengths of PR were measured using ImageJ 1.43 software [36], and the numbers of LR were counted under microscopy. For soil culture, 3-day-old plants, transferred from 1/2 MS media, were grown in a growth chamber with a 16/8 h photoperiod at 22/18 °C, 75% RH and 100 μmol m^−2^ s^−1^ light for 20 days, and then they were irrigated with 150 mM NaCl or 300 mM sorbitol solution once every 3 days. Photos were taken after 15 days of treatment just before harvesting.

### 3.3. Determination of WNK8 Promoter Activity

For detecting *AtWNK8* promoter activity using transgenic plants, 2-day-old or 7-day-old or four-week-old homozygous T3 transgenic plant tissues were directly submerged into GUS staining solution, as described by Jefferson *et al.* (1987) [33], and incubated at 37 °C. The chlorophyll of plant samples was cleared in ethanol, and photographs of GUS activity were taken using a digital camera under a stereomicroscopy (Leica DMB5000). Treated seedlings grown in half strength 1/2 MS media containing NaCl or sorbitol, as indicated, were taken at indicated time points before being photographed.

### 3.4. Determination of *WNK8* Transcript Level under Salt and Osmotic Stress

Seven-day-old plants cultivated in 1/2 MS media were transferred to 1/2 MS media supplemented with NaCl or sorbitol at tested concentrations for the indicated time. Total RNAs were extracted, as described by Wang *et al.* (2008) [13]. qRT-PCR was performed using the ToYoBo qRT-PCR SYBR Green Mix kit (ToYoBo, Japan) with a Corbett Research Rotor-Gene 2000 cycler. The reference housekeeping gene, *AtEF1a* (*At1g07920*), was used as the control and used to normalize qRT-PCR results. Accordingly, the expression level of *AtEF1a* was set as 1.0, the relative expression levels of other genes were determined via the comparison with that of *AtEF1a*. A standard curve was obtained for each gene using different dilutions of cDNA template mix. Reaction conditions for thermal cycling were: 95 °C for 2 min, 40 cycles of 95 °C for 15 s, 56 °C for 15 s and 72 °C for 45 s. Fluorescence data were collected during the cycle at 72 °C. Reactions were technically repeated three times independently. Quantification of each gene was performed using the Corbett Research Rotor-Gene software. Related primers were list in Table S1.

### 3.5. Determination of FW, RWC and Chlorophyll Content

Three-day-old seedlings were germinated in the standard 1/2 MS media containing 150 mM NaCl or 300 mM sorbitol. After 7 days, FWs were measured. Three-day-old seedlings germinated in standard 1/2 MS agar plates were transferred to pots and cultivated for 20 days, then irrigated with 150 mM NaCl or 300 mM sorbitol every three days. After 15-days’ treatment, the chlorophyll was then extracted with 100% aqueous acetone, and total chlorophyll content (including chlorophyll a and b) was measured, as described previously [37]. Six leaves from each plant treated as mentioned above were used for RWC determination; after FW determination, the tissues were placed in distilled water for 24 h at room temperature. The hydrated shoot tissues were weighed to determine the turgid weight (*TW*). The tissues were subsequently dried in an oven at 60 °C for 48 h and weighed to determine the dry weight (*DW*). *RWC* was calculated as *RWC* = (*FW* − *DW*)/(*TW* − *DW*) × 100, as described by Smart (1974) [38].

### 3.6. Measurement of Proline Content and Activities of CAT and POD

Three-day-old seedlings germinated in standard 1/2 MS agar plates were transferred to pots and further cultivated for 20 days, then irrigated with 150 mM NaCl or 300 mM sorbitol every three days. After 15-days’ treatment, plants were harvested for proline, CAT and POD analysis.

Proline content was measured, as described by Bates (1973) [39]. Briefly, about 0.3 g shoots from both control and treatment groups were homogenized with liquid nitrogen. Tissue powders were suspended in 1 mL of 3% sulfosalicylic acid and centrifuged at 1000 × *g* for 5 min at 4 °C. 0.1 mL. The supernatant was mixed with 0.2 mL acid ninhydrin, 0.2 mL 96% acetic acid and 0.1 mL 3% sulfosalicylic acid. The mixtures were incubated at 96 °C for 1 h, mixed with 1 mL toluene and further centrifuged at 1000 × *g* for 5 min at 4 °C. Upper phases were collected, and the absorbencies were read at 520 nm. The proline concentration was determined by using an extinction coefficient of 0.9999 that was derived from a standard curve.

For the activities of CAT and POD, leaf samples from control and stress treatments were homogenized with liquid nitrogen and suspended in phosphate buffer (pH 7.8) for enzyme activity analysis. The suspensions were centrifuged at 12,000 × *g* for 20 min at 4 °C, and the supernatants were used for activity analysis. The protein content in shoot extracts was determined with Bradford’s method [40], using bovine serum albumin as a standard. Catalase (CAT) activity was determined accordingly [41]. Samples containing 100 μg protein were suspended in 1 mL of 50 mM Tris-HCl solution at pH 7.8. The assay medium consisted of 50 mM potassium phosphate buffer at pH 7 and 10 mM H_2_O_2_. The decrease of the H_2_O_2_ absorbance was followed for 90 s at 240 nm at room temperature. Nanomoles of hydrogen peroxide consumed per minute were defined as one unit of CAT. POD activity was determined according to Shannon *et al.* (1966) [42]. The reaction mixture consisted of 3 mL of 0.1 M phosphate buffer (pH 7.0), 0.04 mL of 0.1 M H_2_O_2_, 0.04 mL of 0.2% *O*-dianisidine and a sample containing 25 μg protein. The change in absorbance was recorded at 470 nm for 90 s.

### 3.7. Data Analysis

All the experiments were performed with three to five replicates. All of the data were analyzed with Microsoft Excel 2000 (Microsoft) for calculating mean and standard error (SE). Significances were tested using Student’s *t*-test at *p* < 0.05 level.

## 4. Conclusions

Previous studies have indicated that AtWNK8 interacts with the C subunits of V-ATPase and EDM2, but the roles of *WNK8* in salt and osmotic stresses were unclear before this study. We first provided evidence that increased tolerance of *WNK8* to salt stress is via maintaining higher endogenous activities of CAT and POD; the greater tolerance of *WNK8* to osmotic stress might be dependent on accumulating more proline, along with maintaining higher CAT and POD activities (Figures 2, 3, 4, 5). Our studies shed fresh light on the novel roles of *WNK8* in salt and osmotic stresses and provided new clues to decipher plant networks to abiotic stress responses.

## Supplementary Information



## Figures and Tables

**Table 1:** Bioinformatics analysis of *cis*-elements in the *WNK8* (*At5g14990*) promoter region.

*cis*-Element	Sequence	Position
Phosphorus-related NIT-2 binding site	TATCTA(/G/T)	−1317, −1037, −917
Phosphorus-related TATA box-like binding site	TATAAATA	−797
Light responsive GT-1 binding site	GA(/G)A(/T)AAA(/T)	−1366, −979, −274
Dehydration response DRE binding site	CCGAC	−1546, −1541
Cold response element binding site	ACTCCG	−236
