# Supplementary material for: Disruption of AtWNK8 Enhances Tolerance of Arabidopsis to Salt and Osmotic Stresses via Modulating Proline Content and Activities of Catalase and Peroxidase
Source: Int J Mol Sci. 2013 Mar 27;14(4):7032–47. doi: 10.3390/ijms14047032 (PMC3645675; doi:10.3390/ijms14047032)
Supplement: Supplementary file 1 [file ijms-14-07032-s001.doc]

Supplementary Information

We fused *WNK8* open reading frame (ORF) with the 35S promoter in pCAMBIA1380 and transformed *Arabidopsis* with the floral dipping method. We checked the relative expression levels of *WNK8* in leaves of the four three-week-old transformed lines (T1–T4) with RT-PCR, respectively. Results indicated that the transcription level of *WNK8* in T2 and T4 lines was higher in contrast to the untransformed Colombia ecotype (Control). Hence, we employed T4 as the overexpression line to do further experimentation in this study. Forward primer 5'-ctctgcttgcttcatcaagc-3' and reverse primer 5'-gcgttctctattgcatcctc-3' was used to amplify *WNK8* fragment. The expression level of *WNK8* was normalized to the reference housekeeping gene, *UBQ11*, via qRT-PCR. A partial fragment of *UBQ11* was amplified with primer pairs 5'-gcagattttcgttaaaacc-3' (forward) and 5'-ccaaagttctgccgtcc-3' (reverse).

**Figure S1.** RT-PCR (**a**) and qRT-PCR (**b**) analysis indicated that *AtWNK8* was overexpressed in *Arabidopsis*.

**Table S1.** Primers used for vector construct and quantitative real-time PCR (qRT-PCR).

| **Primer name** | **Sequence (from 5' to 3')** | **Comments** |
| --- | --- | --- |
| AtWNK8OE | For: ACATATATGGCTTCTGGTTCTGGA  Rev: AGAGATGTTAACTGCTTTTTGCTTTTTCG | RT-PCR analysis of *WNK8*  gene expression |
| AtWNK8 | For: GCATTCTATCTGGAGTCAGACAC  Rev: GGTTATGATGCGACGAGGTCC | Quantitative real time PCR analysis of *AtWNK8* transcript level |
| RD29A-qRT | For: ATCACTTGGCTCCACTGTTG TTC  Rev: AAAACACACATAAACATCCAAAGT | Quantitative real time PCR analysis of *RD29A* gene expression |
| AtEF1a-qRT | For: GTCGATTCTGGAAAGTCGACC  Rev: CCGCAATTAATGAGACAGAACA | Quantitative real PCR to check house-keeping gene AtEF1a  transcript level |

© 2013 by the authors; licensee MDPI, Basel, Switzerland. This article is an open access article distributed under the terms and conditions of the Creative Commons Attribution license (http://creativecommons.org/licenses/by/3.0/).
